# *Arabidopsis EGY1* Is Critical for Chloroplast Development in Leaf Epidermal Guard Cells

**DOI:** 10.3390/plants10061254

**Published:** 2021-06-21

**Authors:** Alvin Sanjaya, Ryohsuke Muramatsu, Shiho Sato, Mao Suzuki, Shun Sasaki, Hiroki Ishikawa, Yuki Fujii, Makoto Asano, Ryuuichi D. Itoh, Kengo Kanamaru, Sumie Ohbu, Tomoko Abe, Yusuke Kazama, Makoto T. Fujiwara

**Affiliations:** 1 Department of Materials and Life Sciences, Faculty of Science and Technology, Sophia University, Kioicho, Chiyoda, Tokyo 102-8554, Japan; alvin_sanjaya@protonmail.com (A.S.); ryohsuke67@outlook.com (R.M.); shiho.sato.0815@gmail.com (S.S.); m.suzuki343@gmail.com (M.S.); s.03ch.s@gmail.com (S.S.); hiroki.0831.i@gmail.com (H.I.); y-fujii-5x8@eagle.sophia.ac.jp (Y.F.); m-asano-0h1@eagle.sophia.ac.jp (M.A.); 2 Department of Chemistry, Biology and Marine Science, Faculty of Science, University of the Ryukyus, Okinawa 903-0213, Japan; ryuitoh@sci.u-ryukyu.ac.jp; 3 Faculty of Agriculture, Kobe University, Nada, Kobe 657-8501, Japan; kng@kobe-u.ac.jp; 4 RIKEN Nishina Center, Wako, Saitama 351-0198, Japan; ohbu@riken.jp (S.O.); tomoabe@riken.jp (T.A.); ykaze@fpu.ac.jp (Y.K.); 5 Faculty of Bioscience and Biotechnology, Fukui Prefectural University, Eiheiji, Fukui 910-1195, Japan

**Keywords:** *Arabidopsis thaliana*, At5g35220, epidermal plastid, guard cell, pavement cell, thylakoid

## Abstract

In *Arabidopsis thaliana*, the *Ethylene-dependent Gravitropism-deficient and Yellow-green 1* (*EGY1*) gene encodes a thylakoid membrane-localized protease involved in chloroplast development in leaf mesophyll cells. Recently, *EGY1* was also found to be crucial for the maintenance of grana in mesophyll chloroplasts. To further explore the function of *EGY1* in leaf tissues, we examined the phenotype of chloroplasts in the leaf epidermal guard cells and pavement cells of two ^40^Ar^17+^ irradiation-derived mutants, Ar50-33-pg1 and *egy1-4*. Fluorescence microscopy revealed that fully expanded leaves of both *egy1* mutants showed severe chlorophyll deficiency in both epidermal cell types. Guard cells in the *egy1* mutant exhibited permanent defects in chloroplast formation during leaf expansion. Labeling of plastids with CaMV*35S* or *Protodermal Factor1* (*PDF1*) promoter-driven stroma-targeted fluorescent proteins revealed that *egy1* guard cells contained the normal number of plastids, but with moderately reduced size, compared with wild-type guard cells. Transmission electron microscopy further revealed that the development of thylakoids was impaired in the plastids of *egy1* mutant guard mother cells, guard cells, and pavement cells. Collectively, these observations demonstrate that *EGY1* is involved in chloroplast formation in the leaf epidermis and is particularly critical for chloroplast differentiation in guard cells.

## 1. Introduction

Chloroplasts are semi-autonomous double-membrane-bound organelles containing their own DNA and ribosomes [1,2]. Among the structurally and functionally divergent plastid family members, chloroplasts contain unique, flattened, sac-like thylakoids that carry out oxygenic photosynthesis and release O_2_ gas into the atmosphere, while converting atmospheric CO_2_ into carbohydrates to support plant growth and development [3]. In leaf tissues, chloroplasts are generally distributed in mesophyll, bundle sheath, and epidermal guard cells. Chloroplasts are also present in epidermal pavement cells in many species [2,4,5]. Mesophyll chloroplasts are physiologically important, easily accessible for biochemical and physiological analyses, and therefore have been the primary subject of chloroplast development-related research to date [6,7,8]. By contrast, non-mesophyll chloroplasts have rarely been the target of chloroplast-focused studies.

The leaf epidermis is composed of stomatal guard cells, pavement cells, and trichome cells, which may or may not contain chloroplasts depending on the plant species. Basic information on the structure and intracellular morphology of epidermal chloroplasts in the growing leaves of wild-type plants has been collected mainly from two species, tobacco (*Nicotiana tabacum*) and *Arabidopsis thaliana* [4,5,9,10,11,12,13,14,15,16,17,18,19], although studies on the control of stomatal opening and closing in several model plants have also generated a vast amount of data on guard cell chloroplasts [3,20,21,22,23]. Similarly to mesophyll chloroplasts, pavement and guard cell chloroplasts develop thylakoids (which form stroma lamellae and grana and synthesize chlorophyll pigments), accumulate starch grains in the stroma, and express nuclear- and chloroplast-encoded photosynthetic genes. However, epidermal chloroplasts are smaller, contain fewer thylakoids, and extend more and longer stromules than mesophyll chloroplasts [5,9,19,24,25,26,27,28]. Guard cell chloroplasts preferentially accumulate starch grains via a regulatory mechanism that is distinct from that employed by mesophyll cell chloroplasts [10,23]. Furthermore, the chloroplast phenotypes of representative chloroplast division mutants differ significantly between leaf mesophyll cells and pavement or guard cells, indicating tissue- or cell-type-dependent control of chloroplast division in leaves [17,27,28,29,30,31,32,33]. These examples raise a fundamental question about the control of chloroplast development in the leaf epidermis, or, more specifically, whether chloroplast biogenesis-related factors in leaf mesophyll cells play an equivalent role in the leaf epidermis [34,35,36]. Several recent studies have attempted to address this question. For example, Barton et al. [26] performed imaging analyses to detect different sized epidermal leucoplasts and chloroplasts in the white and green leaf sectors of the *Arabidopsis* variegation mutant, *immutans* (*im*) [37,38], respectively. Negi et al. [39] isolated the *Arabidopsis green less stomata 1* (*gles1*) mutant, which contains a defect in the chloroplast envelope-localized TGD5 protein [40] and exhibits impaired chloroplast development and lipid metabolism in leaf guard cells but not in mesophyll cells. More recently, Itoh et al. [41] reported a new mutant, *stromule biogenesis altered1* (*suba1*; allelic to *tgd5*), which exhibits abnormalities in chloroplast development and autophagic processes in guard cells. These studies suggest a fundamentally conserved, but differentially tuned, regulation of leaf epidermal and mesophyll chloroplasts. However, regulation of epidermal chloroplasts remains largely elusive, and additional efforts are needed to better understand chloroplast development.

*Ethylene-dependent Gravitropism-deficient and Yellow-green 1* (*EGY1*), a member of the SREBP S2P protease gene family, encodes a thylakoid membrane-targeted metalloprotease [42]. Originally, *EGY1* was identified in an *Arabidopsis* mutant that showed defective ethylene-dependent hypocotyl gravitropism and leaf greening phenotypes. The predicted EGY1 protein contains eight transmembrane domains and an N-terminal transit peptide sequence, and it exhibits ATP-independent proteolytic activity in vitro. Under normal growth conditions, the *egy1* mutant produces pale leaves, with grana-less mesophyll chloroplasts [42]. Interestingly, the *egy1* mutant exhibits hypersensitivity to exogenous application of high ammonium concentrations [43], leaf chlorosis in association with elevated expression of several senescence-associated marker genes (e.g., *SAG12* and *SEN4*) [44], and enhanced leaf variegation phenotype in the *var2-5* mutant background [45]. Notably, similar chlorotic phenotypes have been reported in mutants of the orthologous genes from several plant species, including tomato (*Solanum lycopersicum*) [46], *Setaria italica* [47], and *N. tabacum* [48]. This suggests that *Arabidopsis EGY1* and its orthologs in other plant species play important roles in leaf chloroplast biogenesis and maintenance, although their contribution to these processes may vary among species.

Recently, we isolated an argon ion (^40^Ar^17+^)-irradiated pale green *Arabidopsis* mutant, Ar50-33-pg1 [49], which carried a single large deletion (940,000 bp) on chromosome 5, encompassing over 40 protein-coding genes, including *EGY1*. We showed that both Ar50-33-pg1 and another *egy1* mutant, *egy1-4* [50], exhibited preferential degeneration of mesophyll chloroplast grana, along with progression of leaf chlorosis [49], suggesting that *EGY1* is critical for the maintenance of chloroplasts in leaf mesophyll cells. During mutant screening, we noticed chlorophyll deficiency in leaf stomatal guard cells of Ar50-33-pg1, which led us to examine the process of chloroplast formation in the leaf epidermis of Ar50-33-pg1. Here, we characterize the two loss-of-function mutants of *EGY1*, and, extending our previous findings [49], reveal the critical involvement of *EGY1* in the formation of chloroplasts in guard cells and pavement cells.

## 2. Results

### 2.1. Chlorophyll Deficiency in the Epidermis of Expanded Leaves of Ar50-33-pg1 and egy1-4 Mutants

**Figure 1:**
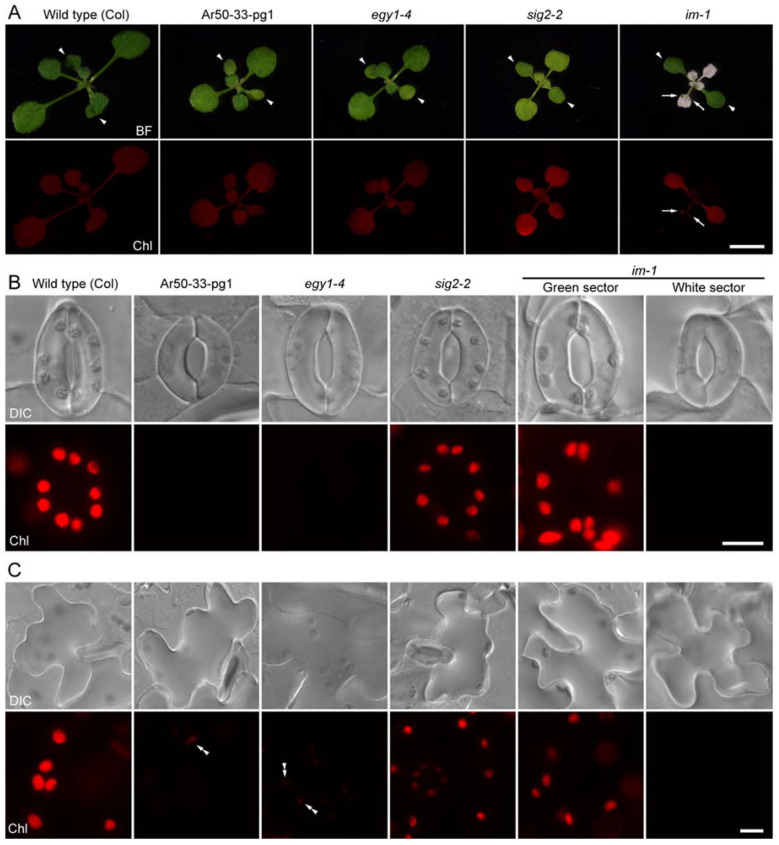
Fluorescence microscopy of the epidermis of fully expanded leaves of wild-type and mutant plants. (**A**) Photographs of 2-week-old wild-type (Col), Ar50-33-pg1, *egy1-4*, *sig2-2*, and *im-1* seedlings grown in soil. Arrowheads and arrows indicate cotyledons and green sectors in variegated leaves, respectively. (**B**,**C**) Differential interference contrast (DIC) and chlorophyll autofluorescence (Chl) images of guard cells (**B**) and pavement cells (**C**) in the abaxial leaf epidermal peels of the primary leaves of 3-week-old seedlings. Double arrowheads in (**C**) indicate chloroplasts. Chl images in (**B**,**C**) were captured under the same microscopic conditions. Bar = 5 mm (**A**) and 10 µm (**B**,**C**).

During the screening process of Ar50-33-pg1 [49] (Figure 1A), we surveyed defects (other than mesophyll chlorosis) in leaf chloroplast formation. Epifluorescence microscopy analysis of abaxial leaf epidermal peels revealed severe chlorophyll deficiency in the stomatal guard cells of expanded leaves (Figure 1B). Pavement cells also exhibited chlorophyll deficiency (Figure 1C). The severity of chlorophyll deficiency varied moderately among stomata (paired guard cells) and among pavement cells, but guard cells generally exhibited more severe defects than pavement cells. Although rarely, the pavement cells exhibited wild type-like levels of chlorophyll fluorescence. By contrast, none of the guard cells showed a wild type-like phenotype (data not shown).

Generally, the epidermis of expanded wild-type leaves contained spherical to ellipsoidal chloroplasts, with a diameter of 3–4 µm in guard cells and 4–7 µm in pavement cells. These results were consistent with the fluorescence and confocal microscopy observations reported previously [5,15,16,26,30]. In the leaf epidermis of Ar50-33-pg1, however, little or no chlorophyll fluorescence was detected under normal fluorescence microscopy conditions, and, in most cases, chlorophyll fluorescence signals were barely detected under the maximal strength of excitation and detection conditions (Figure S1). Additionally, it was very difficult to determine the number and size of Ar50-33-pg1 chloroplasts or plastids because of the faint fluorescence signals and heterogeneity within and between cells (data not shown).

A reciprocal backcross between Ar50-33-pg1 and Col confirmed that chlorophyll deficiency in the epidermis of Ar50-33-pg1 was caused by a single recessive mutation, which was attributed to the large (940 kb) deletion [49]. To determine whether the epidermal chlorophyll defects were caused by the deletion of *EGY1* in Ar50-33-pg1, we characterized another *egy1* mutant, *egy1-4* (Figure 1A), which carries a 5 bp deletion in exon 4 and a 5 bp substitution in the intron 3–exon 4 region of *EGY1* [50]. Our segregation analysis (Table S1), rough mapping (by crossing with L*er*) [51], and RT-PCR experiments [52] indicated that *egy1-4* is a loss-of-function allele that produces C-terminally truncated but N-terminally intact proteins due to incorrect splicing and frameshift mutation [52]. Microscopic analysis of *egy1-4* mutant leaves revealed the presence of achlorophyllous guard cells and pavement cells, similar to those in Ar50-33-pg1 leaves (Figure 1B,C and Figure S1). Moreover, an allelic test between Ar50-33-pg1 and *egy1-4* mutants showed that the F_1_ progeny of both mutants exhibited leaf epidermal chlorophyll deficiency similar to that observed in the parental genotypes (Figure S2), along with the pale phenotype [49]. We further corroborated these results by analyzing the T-DNA insertion (null) mutant, *egy1-2* [42]. Both *egy1-2* and its F_1_ progeny (derived from the *egy1-2* × *egy1-4* cross) showed phenotypes similar to those of *egy1-4* in terms of leaf chlorosis and epidermal chlorophyll deficiency (Figure S3). Therefore, we concluded that the loss-of-function mutation of *EGY1* led to chlorophyll deficiency in the leaf epidermis.

To examine whether the epidermal chloroplast defects in *egy1* could be generally applied to other chloroplast biogenesis mutants, we examined the *sig2-2* [53,54] and *im-1* [37] mutants, as references. The *sig2-2* mutant exhibits the virescent leaf phenotype, while the *im-1* mutant shows the variegation phenotype (Figure 1A). Although young leaves of the *sig2-2* mutant were paler than those of Ar50-33-pg1 and *egy1-4* mutants, *sig2-2* leaves exhibited a relatively modest reduction in chlorophyll fluorescence in both guard cells and pavement cells (Figure 1B,C). In the *im-1* mutant, while the green leaf sectors contained both chlorophyll fluorescence signal-positive and -negative pavement and guard cells, the white leaf sectors mostly contained achlorophyllous pavement and guard cells (Figure 1B,C; see also [55]). Although in-depth characterizations are required to establish the various phenotypes, these results imply that the epidermal chloroplast phenotype of mesophyll chloroplast biogenesis mutants cannot be deduced easily. The loss-of-function of EGY1 seemed to affect chloroplast formation in the leaf epidermis, with the defect being more severe in guard cells than in pavement cells.

### 2.2. Conservation of Epidermal Chlorophyll Deficiency in Expanding Leaves of Ar50-33-pg1 and egy1-4 Mutants

*EGY1* is a positive regulator of chloroplast differentiation in leaf mesophyll cells [42,45], and its loss-of-function induces rapid grana disassembly therein [49]. Epidermal chlorophyll deficiency in expanded *egy1* leaves might be accompanied by impaired chloroplast development, chloroplast degeneration after differentiation, or both. We examined this possibility by performing epifluorescence microscopy analysis of the third and fourth leaves (length: 3–5 mm) of 2-week-old wild-type, Ar50-33-pg1, and *egy1* seedlings (Figure 1A).

**Figure 2:**
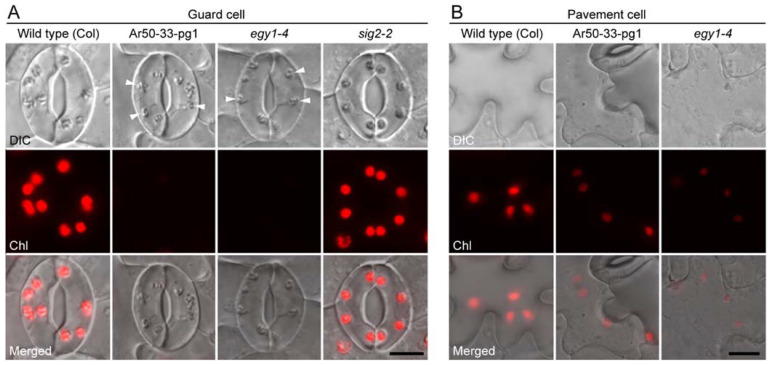
Fluorescence microscopy of the epidermis of expanding wild-type and mutant leaves. (**A**,**B**) DIC, chlorophyll autofluorescence (Chl), and merged images of guard cells (**A**) and pavement cells (**B**) in the abaxial leaf epidermal peels of the third and fourth leaves of 2-week-old seedlings. Arrowheads indicate putative chloroplasts or plastids. Bar = 10 µm.

Despite their young developmental stage, the wild-type leaves contained well-developed chloroplasts in mature guard cells. However, no (or barely detectable) chlorophyll fluorescence was observed in Ar50-33-pg1 and *egy1-4* guard cells (Figure 2A). The severe attenuation of the chlorophyll fluorescence signal in the guard cells of both mutants was similar to the phenotype of their expanded leaves and was more pronounced than that in phenotype *sig2-2*, in which guard cells contained developed chloroplasts (Figure 2A).

Differentiated chloroplasts, if not fully developed, were observed in the pavement cells of wild-type expanding leaves. The pavement cells showed impaired development of chloroplasts, in terms of both chloroplast size and chlorophyll fluorescence level, in Ar50-33-pg1 and *egy1-4* mutants. These chloroplast defects in pavement cells were less severe than those observed in guard cells in both mutants (Figure 2B), and pavement cells showed greater variation in the degree of chloroplast defects than guard cells in both young and mature leaves. However, the nature of chloroplast defects was conserved between expanded (Figure 1C) and expanding (Figure 2B) leaves.

During microscopy analyses, we also noticed the presence of pigment-less spherical substructures in guard cells (Figure 2A, arrowheads). The intracellular localization pattern and size of these structures resembled those of chloroplasts [15] (Figure 1 and Figure 2). Therefore, these structures were considered to represent starch-accumulating chlorophyll-less plastids. 

Overall, while mutations in *EGY1* permit chloroplast differentiation in leaf mesophyll cells [49], these mutations prevented chloroplast differentiation in guard cells and pavement cells. Additionally, mutations in *EGY1* resulted in modest, but distinct, defects in chloroplast development between guard cells and pavement cells. Chloroplast differentiation was strongly inhibited in guard cells, but weakly or partially inhibited in pavement cells. No particular differences in the epidermal chloroplast phenotypes were detected between Ar50-33-pg1 and *egy1-4* mutants.

### 2.3. Generation of Transgenic Arabidopsis Lines with Green Fluorescent Protein (GFP)-Labeled Plastids

Labeling of stroma with fluorescent proteins is a convenient and reliable method for imaging plastids in live plant tissues [11,18,24,25]. The strong and constitutive cauliflower mosaic virus *35S* promoter (CaMV*35S*) [56] has been utilized mostly to this end. However, to the best of our knowledge, shoot epidermis-specific promoters [57,58] have never been tested, except for investigating the role of the epidermis in the development of mesophyll chloroplasts [34]. Young epidermal tissues are generally difficult to isolate in an intact and pure form [20].

**Figure 3:**
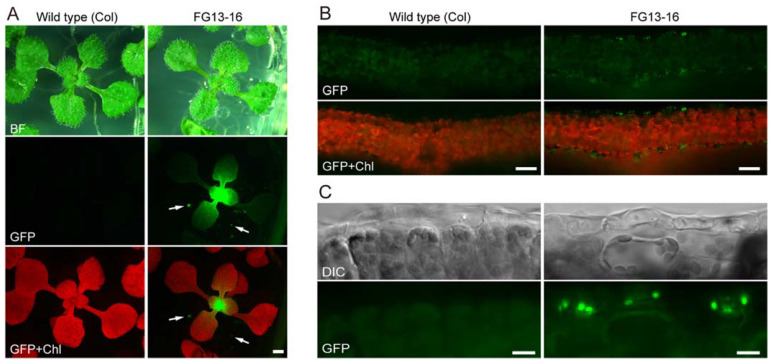
Fluorescence stereomicroscopy and epifluorescence microscopy analyses of 2-week-old wild-type (Col) and *PDF1*p::*TP_SIG6_-GFP* transgenic (FG13-16) *Arabidopsis* seedlings grown on Murashige and Skoog (MS) medium. (**A**) Plant growth. Arrows indicate the local accumulation of GFP in transgenic roots. (**B**,**C**) Cross-sections of leaves. DIC, GFP, or chlorophyll autofluorescence (Chl) images or the merged GFP and Chl images are shown at low (**B**) and high magnification (**C**). Bar = 1 mm (**A**), 50 µm (**B**), and 10 µm (**C**).

We generated transgenic *Arabidopsis* lines expressing the plastid sigma factor 6 (SIG6) transit peptide (TP)-fused synthetic (S65T) *GFP* (*TP_SIG6_-GFP*) gene [59,60] under the shoot epidermis-specific *protodermal factor1* (*PDF1*) promoter [61,62]. The *PDF1*p::*TP_SIG6_-GFP* construct was introduced into wild-type (Col) plants via *Agrobacterium*-mediated transformation [63]. Several hundred T_1_ seedlings were obtained by screening for bialaphos resistance conferred by the T-DNA. Among these transformants, relatively strong GFP-positive seedlings were chosen for further screening (Figure S4). The GFP signal intensity, and its localization in tissues, varied moderately among the transgenic lines. However, transgenic lines tended to accumulate GFP at the shoot apex, leaf primordia, and in expanding leaves, forming a fluorescence gradient, which decreased from young to old tissues along the developmental axis (Figure 3A and Figure S4). Finally, the transgenic line FG13-16, which exhibited high and stable GFP fluorescence over three generations, was selected for analysis. GFP localization in the leaf epidermal chloroplasts of FG13-16 was confirmed in young leaves and leaf primordia by epifluorescence microscopy (Figure 3B and Figure S5A, and data not shown). These expression patterns of GFP in the leaf epidermis were consistent with the *PDF1* promoter activity in shoot protodermal and epidermal tissues reported previously [61,62].

The transgenic plants also showed a GFP signal in roots (Figure 3A, Figures S4 and S5B), which was localized at the primordia and tip of lateral roots. Under high magnification, GFP-labeled proplastids in primordial cells appeared as discrete intracellular organelles with diverse shapes (globular, amoeboid, and filamentous (Figure S5C)), indicating that these transgenic lines could be used to study plastids during root development. During the reproductive phase, GFP accumulated at the shoot apex (similar to that during the vegetative phase) and the surrounding floral organs (Figure S5D). GFP was also detected in the embryos of siliques (Figure S5E–G). Our preliminary observations, however, indicated that these shoot apex- and embryo-localized GFP signals existed not only in the outermost epidermal tissues but also in internal tissues (data not shown), cautioning that the selection of lines exhibiting strong GFP signals, and therefore efficient chloroplast labeling, did not necessarily reflect the authentic expression of the reference gene (i.e., *PDF1*) [61,62,64,65] in several tissues. Nevertheless, we concluded, in accordance with the objective of this study, that a plastid-labeled marker line for studying plastids in the epidermis of young shoots was obtained successfully.

### 2.4. Visualization of Chlorophyll-Deficient Plastids in the Leaf Epidermis of Ar50-33-pg1 and egy1-4 Mutants

To generate plastid-labeled lines in the *egy1* mutant background, we crossed the FG13-16 transgenic line as the male parent with Ar50-33-pg1 or *egy1-4* (female parent). In parallel with this experiment, another transgenic line, FL6-5, expressing both *AtFtsZ1-1 TP-fused Yellow Fluorescent Protein* gene (*TP_FtsZ1_-YFP*) and *Cryptochrome 2* (*Cry2*) *nuclear localization signal* (*NLS*)-fused *Cyan Fluorescent Protein* gene (*NLS_Cry2_-CFP*) under the control of the constitutive CaMV*35S* promoter [32], was crossed with both mutants to obtain another set of fluorescent mutants. The FG13-16-derived lines facilitated the analysis of epidermal chloroplasts in expanding leaves, while the FL6-5-derived lines facilitated the detection of epidermal chloroplasts in expanded leaves with the intense YFP signal.

**Figure 4:**
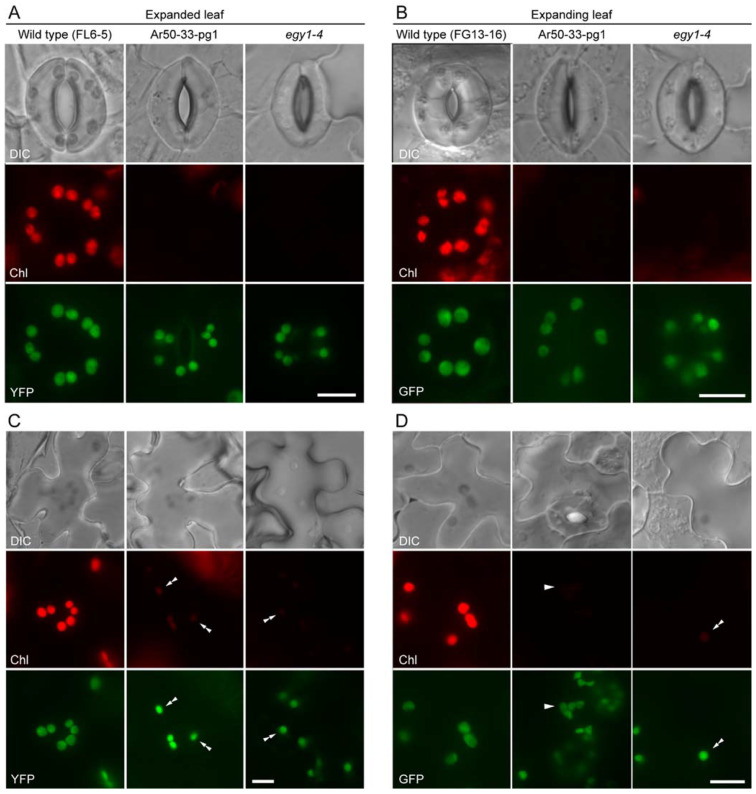
Fluorescence microscopy analysis of leaf epidermis in the wild type and in mutant lines expressing stroma-targeted fluorescent proteins. (**A**,**B**) Guard cells in fully expanded (**A**) and expanding (**B**) leaves. (**C**,**D**) Pavement cells in expanded (**C**) and expanding (**D**) leaves. In (**A**,**C**), DIC, chlorophyll autofluorescence (Chl) and YFP images of the primary leaves of 3-week-old wild-type (FL6-5), Ar50-33-pg1 (Ar50-33-pg1 × FL6-5), and *egy1-4* (*egy1-4* × FL6-5) transgenic seedlings are shown, while in (**B**,**D**), DIC, Chl, and GFP images of the third and fourth expanding leaves of 18-day-old wild-type (FG13-16), Ar50-33-pg1 (Ar50-33-pg1 × FG13-16), and *egy1-4* (*egy1-4* × FG13-16) transgenic seedlings are shown. Double arrowheads in (**C**,**D**) indicate the presence of chloroplasts. Arrowheads in (**D**) correspond to those in Figure S6 and indicate pavement cell chloroplasts. Chl images in (**A**–**D**) were captured under the same microscopic conditions. Bar = 10 µm.

Guard cells in both expanded and expanding leaves of Ar50-33-pg1 and *egy1-4* mutant contained green fluorescent structures, which were spatially merged with the faint chlorophyll fluorescence signals, confirming that these structures were chloroplasts (plastids) (Figure 4A,B, and data not shown). Notably, despite the severe loss of chlorophyll fluorescence, the configuration and intracellular distribution of mutant chloroplasts or plastids in guard cells were largely consistent with those of wild-type chloroplasts. The only difference between wild-type and mutant guard cell plastids, other than the remarkable chlorophyll reduction in mutant plastids, was the modest size reduction in *egy1* plastids. Pavement cells in the leaves of both *egy1* mutants also exhibited similar results (Figure 4C,D). Green fluorescent bodies, which merged perfectly with regions emitting chlorophyll fluorescence signals, exhibited a scattered distribution in mutant cells, similar to that observed in wild-type cells (Figure 4C,D, double arrowheads). These pavement cell chloroplasts or plastids in Ar50-33-pg1 and *egy1-4* were only moderately reduced in size relative to those in the wild type.

To evaluate the effects of mutations in *EGY1* on the replication of epidermal cell chloroplasts or plastids, we counted the number of GFP-positive plastids in mature guard cells of *PDF1*p::*TP_SIG6_-GFP*-expressing wild-type (FG13-16), Ar50-33-pg1 (Ar50-33-pg1 × FG13-16), and *egy1-4* (*egy1-4* × FG13-16) seedlings (Table 1). The third and fourth leaves of 18-day-old FG13-16, Ar50-33-pg1 × FG13-16, and *egy1-4* × FG13-16 seedlings were examined by epifluorescence microscopy. The results showed that the number of guard cell plastids in Ar50-33-pg1 and *egy1-4*, both at the single-cell level and stomatal (guard cell pair) level, were similar to that in their wild-type counterparts (data were not significantly different (*p* > 0.05) between samples by the Kruskal–Wallis test). This result was in marked contrast to the previously reported results for *arc6* [30,32] and *atminE1* [32] mutants, in which non-photosynthetic plastids were capable of massive proliferation in a population of chlorophyll-deficient guard cells, overcoming defects in the chloroplast division machinery, although the underlying mechanism remains unknown. Thus, the shape and number of plastids were unaffected by the absence of chlorophyll in *egy1* guard cells, suggesting that the proliferation of chloroplasts does not require a functional EGY1 protein in the leaf epidermis, and the regulatory mechanisms that determine the photosynthetic competence of chloroplasts and the control of chloroplast division can be separated during leaf formation.

### 2.5. Conserved Chlorophyll-Deficient Phenotype of Cotyledon Guard Cells of Ar50-33-pg1 and egy1-4 Mutants

We extended our characterization of *egy1* mutant guard cells to that of cotyledons. Cotyledons are developed from embryonic cells in seeds, rather than from the shoot apical meristem [3,66], and develop chloroplasts in mesophyll cells, guard cells, and pavement cells (as in leaves). Chloroplast development in cotyledons is believed to be controlled differently from that in leaves, based on the identification of mutants with organ-specific abnormalities in chloroplast biogenesis (e.g., [67,68,69]). Therefore, the status of cotyledon chloroplasts in *egy1* mutants could be of interest.

**Figure 5:**
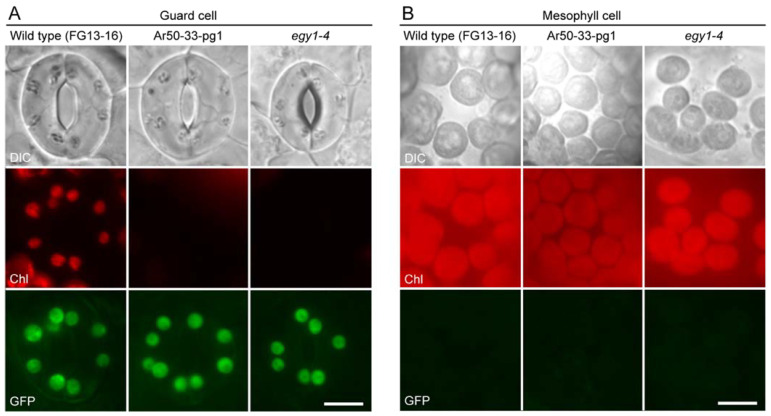
Fluorescence microscopy of cotyledon epidermis in the wild-type and mutant lines expressing stroma-targeted fluorescent proteins. (**A**,**B**) DIC, chlorophyll autofluorescence (Chl), and GFP images of guard cells (**A**) and mesophyll cells (**B**) in the abaxial epidermal peels of cotyledons of 1-week-old seedlings. Bar = 10 µm.

Guard cells in the mature cotyledons of Ar50-33-pg1 × FG13-16 and *egy1-4* × FG13-16 lines exhibited severe chlorophyll deficiency (Figure 5A). The *PDF1* promoter in FG13-16 expressed the *TP_SIG6_-GFP* fusion specifically in the cotyledon epidermis, which confirmed the preservation of plastids, as in leaf guard cells. No noticeable differences in the size of plastids could be observed between the wild type and *egy1* mutants (Figure 5A). On the other hand, mesophyll chloroplasts in cotyledons of the wild-type and mutant lines lacked GFP and seemed to show normal differentiation, based on the chloroplast size and chlorophyll fluorescence (Figure 5B). Although the degeneration phenotype of cotyledon mesophyll chloroplasts was not established and requires substantial inspection in the future, our results indicate that *EGY1* is crucial for the formation of guard cell chloroplasts in cotyledons.

### 2.6. Chlorophyll Deficiency in Developing Guard Cells in the Leaf Epidermis of Ar50-33-pg1 and egy1-4 Mutants

Stomatal development during leaf expansion involves the unique processes of cell division and expansion through the formation of several progenitor cell types [70]. This program is not strictly limited to the proliferation and differentiation zones of mesophyll cells in the leaf [71,72]. Guard mother cells represent a direct precursor of paired guard cells and contain chloroplasts [10]. While the mesophyll chloroplast development program is generally thought to follow a developmental gradient of leaves along the distal–proximal axis [6,72,73,74], the development of epidermal chloroplasts, including that of guard cells, remains largely unknown. A significant expansion of chloroplasts (plastids) occurs during the formation of guard cells (length: 3–4 µm) from guard mother cells (length: 1–2 µm) [10] (Figure 1B and Figure 4A,B). We therefore examined the existence and morphology of chloroplasts (plastids) in guard mother cells using expanding leaves of FG13-16 (*PDF1*p::*TP_SIG6_-GFP*-expressing wild type), Ar50-33-pg1 × FG13-16, and *egy1-4* × FG13-16 plants.

**Figure 6:**
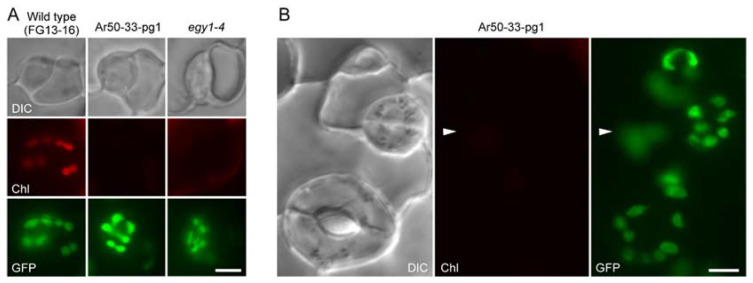
Fluorescence microscopy of leaf guard mother cells in the wild-type and mutant lines expressing *PDF1*p::*TP_SIG6_-GFP*. (**A**,**B**) DIC, chlorophyll autofluorescence (Chl), and GFP images of guard mother cells (**A**) and late stomatal lineage cells (**B**) in the abaxial leaf epidermal peels of the third and fourth expanding leaves of 18-day-old seedlings. Arrowheads in (**B**) correspond to those in Figure 4 and Figure S6 and indicate pavement cell chloroplasts. Chl images in (**A**,**B**) were captured under the same microscopic conditions. Bar = 5 µm.

Guard mother cells in wild-type leaves exhibited chlorophyll autofluorescence in small-sized chloroplasts (Figure 6A). These chloroplasts were frequently highly elongated, with a constriction at the mid-point. Based on our previous observation of FtsZ1 ring-mediated chloroplast division in guard cells of expanding leaves [75], these elongated chloroplasts were predicted to be undergoing binary fission [76]. However, guard mother cells in both Ar50-33-pg1 and *egy1-4* leaves exhibited no (or barely detectable) chlorophyll autofluorescence. Instead, GFP-labeled plastids, with a normal size and shape, were observed (Figure 6A,B and Figure S6), which coincided with the mature guard cells in expanded and expanding leaves of both Ar50-33-pg1 and *egy1-4* mutants. Thus, the loss-of-function of *EGY1* led to chlorophyll deficiency in leaves during the development of stomatal guard cells (Figure 6B and Figure S6B).

### 2.7. Ultrastructure of Guard Cell Plastids in the Leaf Epidermis of Ar50-33-pg1 and egy1-4

**Figure 7:**
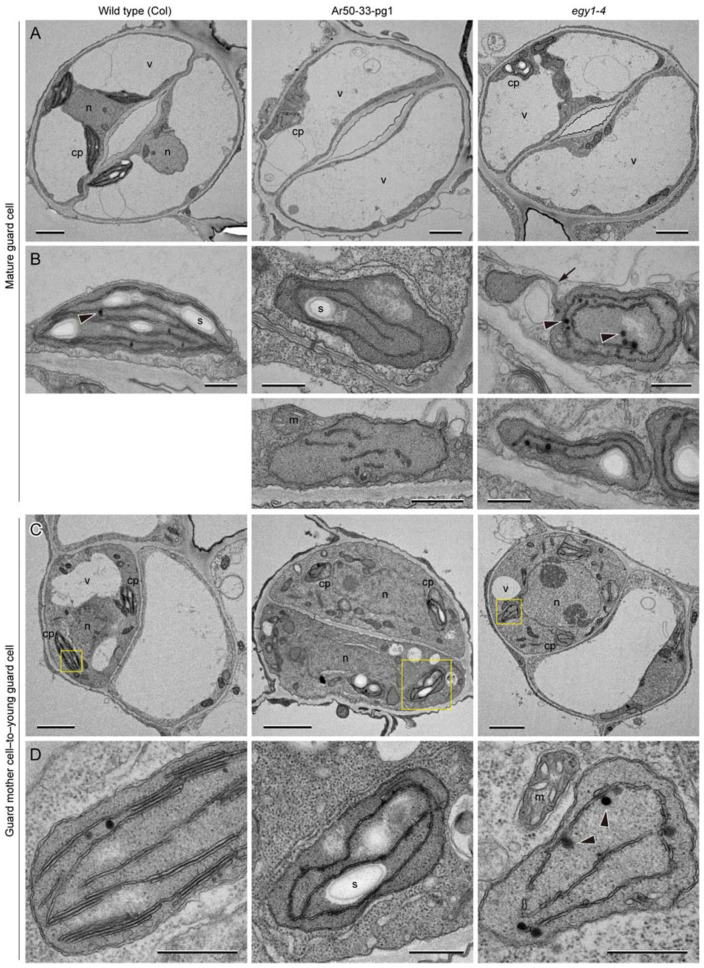
TEM analysis of guard cells and guard mother cells in expanding leaves of wild-type and mutant plants. Primary leaves of 10-day-old wild-type (Col), Ar50-33-pg1, and *egy1-4* plants were analyzed. (**A**,**B**) Mature guard cells (**A**) and chloroplasts therein (**B**). (**C**,**D**) Guard mother cells dividing into two guard cells (**C**), and chloroplasts therein (**D**). Boxed regions in (**C**) are magnified in (**D**) (the image is rotated). Arrows and arrowheads indicate stromules and plastoglobules, respectively. cp, chloroplast; n, nucleus; s, starch; v, vacuole. Bar = 2 µm (**A**,**C**) and 0.5 µm (**B**,**D**).

**Figure 8:**
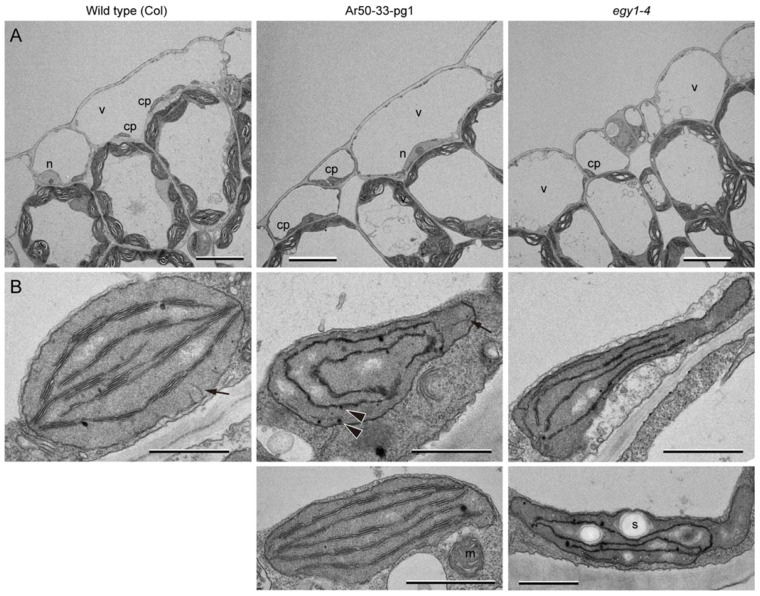
TEM analysis of pavement cells in the expanding wild-type and mutant leaves. Primary leaves of 10-day-old wild-type (Col), Ar50-33-pg1, and *egy1-4* plants were analyzed. (**A**) Pavement cells. (**B**) Pavement cell chloroplasts. Arrows and arrowheads indicate tubular structures extending from the inner envelope and plastoglobules, respectively. See also Figure S7. Abbreviations: cp, chloroplast; n, nucleus; s, starch grain; v, vacuole. Bar = 10 µm (**A**) and 1 µm (**B**).

**Figure 9:**
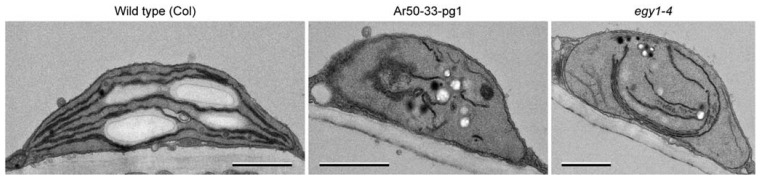
TEM analysis of pavement cells in the fully expanded leaves of wild-type and mutant plants. Primary leaves of 3-week-old wild-type (Col), Ar50-33-pg1, and *egy1-4* plants were analyzed. Bar = 1 µm.

To investigate the plastid ultrastructure in the chlorophyll-deficient leaf epidermis of Ar50-33-pg1 and *egy1-4* mutants, we examined the abaxial epidermis of the primary leaves of 10-day- and 3-week-old wild-type (Col), Ar50-33-pg1, and *egy1-4* seedlings by transmission electron microscopy (TEM) (Figure 7, Figure 8 and Figure 9), as in our recent analyses of mesophyll chloroplasts [49]. Mesophyll cells in 10-day-old plant leaves are expected to occur exclusively at the chloroplast building stage, while those in 3-week-old plants leaves are expected to occur at the stage of chloroplast maintenance to early destruction.

The overall configurations of mature guard cells in both Ar50-33-pg1 and *egy1-4* mutants appeared similar to those of the wild type (Figure 7A). The structures of central vacuoles, nuclei, and cell walls in both mutants were also similar to those in the wild type, in accordance with previous observations [10]. At the subcellular level, guard cells in Ar50-33-pg1 and *egy1-4* leaves contained chloroplasts that produced starch grains similar to the wild-type guard cells; however, the mutant guard cells exhibited defects in the thylakoid structure. Wild-type guard cell chloroplasts contained typical thylakoid systems composed of up to five stacks of granal thylakoids and stromal lamellae running from pole to pole along the longitudinal axis of chloroplasts. By contrast, mutant guard cell chloroplasts contained single-layered thylakoids without grana stacking (Figure 7B). These ‘stroma lamellae’-like thylakoids were frequently detected in not only long filamentous forms but also spiral, circular, and short filamentous forms in nearly normal-sized chloroplasts. Moreover, an abnormally large number of electron-dense plastoglobules were detected in mutant chloroplasts. The tubular stroma-containing substructures of plastids, stromules, were occasionally detected in guard cells of both wild-type and *egy1* leaves (Figure 7B, and data not shown).

Next, we focused on guard mother cells and young guard cells. The guard mother cells in wild-type leaves developed chloroplasts containing up to five stacks of granal thylakoids (Figure 7C,D). Additionally, the wild-type chloroplasts in guard mother cells accumulated starch grains, consistent with the previous report [10]. Guard mother cells and young guard cells in Ar50-33-pg1 and *egy1-4* mutant leaves were indistinguishable from those in wild-type leaves, except for the chloroplast interior structure. Chloroplasts in both Ar50-33-pg1 and *egy1-4* mutants contained only single-layered thylakoids in most regions. These thylakoids were occasionally twisted, and two adjacent single-layered thylakoids formed a short attachment site or an interconnection. Additionally, although the *egy1* mutant chloroplasts could produce starch grains, aberrant thylakoid forms (spirals, circles, or short filaments) were observed, as in the mature guard cell (Figure 7C,D and data not shown).

### 2.8. Ultrastructure of Pavement Cell Plastids in the Leaf Epidermis of Ar50-33-pg1 and egy1-4 Mutants

Next, we examined pavement cells in wild-type and mutant leaves. The overall configurations of pavement cells in Ar50-33-pg1 and *egy1-4* mutant leaves were similar to those of pavement cells in wild-type leaves (Figure 8A), which is consistent with the phenotypes of guard cells and mesophyll cells observed in ultrathin leaf sections (Figure 7) [49]. Compared with the guard cell chloroplasts, pavement cell chloroplasts in Ar50-33-pg1 and *egy1-4* leaves exhibited greater variations in thylakoid structure. Only single-layered thylakoids to the wild type-like multi-layered (granal) thylakoids were found (Figure 8B and Figure S7, and data not shown). Additionally, the mutant pavement cell chloroplasts contained many plastoglobules and exhibited reduced thickness at the whole organelle level, similar to the phenotype of *egy1* mutant guard cell chloroplasts (Figure 7B,D). By contrast, the wild-type pavement cell chloroplasts exhibited a complete lens-like shape, with the basic composition of grana and stromal lamellae. The presence of relatively well-developed chloroplasts in the pavement cells of Ar50-33-pg1 and *egy1-4* leaves (Figure 8B) supported the data obtained by epifluorescence microscopy in this study.

Finally, we examined the epidermal chloroplasts in the leaves of 3-week-old wild-type, Ar50-33-pg1, and *egy1-4* seedlings (Figure 9 and Figure S8). Guard cells and pavement cells in wild-type leaves contained chloroplasts with well-developed thylakoids. The accumulation of starch grains, a characteristic feature of guard cell chloroplasts [10,22,23], was also confirmed (Figure S8). By contrast, the *egy1* mutant guard cell and pavement cell chloroplasts contained heavily damaged or disorganized thylakoids (Figure 7 and Figure 8 and Figure S7). Typically, *egy1* thylakoids lacked grana and did not orient to the chloroplast poles, but instead assumed the extended single-layered, spiral, circular, or short filamentous forms. Multi-layered thylakoids with certain intervals were also occasionally formed. Chloroplasts in *egy1* mutant leaves showed some structural differences, depending on plant age; chloroplasts in the leaves of 3-week-old mutant plants contained more plastoglobules and damaged thylakoids compared with chloroplasts in those of 10-day-old mutant plants.

Taken together, the results of ultrastructure analysis (Figure 7, Figure 8 and Figure 9) supported our fluorescence microscopy observations of chloroplasts in guard cells and pavement cells (Figure 1, Figure 2, Figure 4 and Figure 6, Figures S1 and S6). The remarkable reduction in chlorophyll fluorescence in Ar50-33-pg1 and *egy1-4* mutants was associated with defects in thylakoid structure. Moreover, the relatively broad chlorophyll deficiency phenotypes of *egy1* mutant pavement cells may correspond to the degree of structural anomalies in their thylakoids. Throughout the fluorescence and electron microscopy analyses, the epidermal chloroplast phenotypes of Ar50-33-pg1 and *egy1-4* mutants were indistinguishable. In conclusion, the results of our current mutant analyses indicate that *EGY1* is involved in the proper organization of thylakoids in epidermal cell chloroplasts, and that this involvement is critical for the formation of guard cell chloroplasts during stomata development.

## 3. Discussion

In this study, following the initial characterization of a heavy-ion-induced pale green mutant, Ar50-33-pg1 [49], we investigated the role of a chloroplast protein-coding gene, *EGY1* [42], in leaf epidermal chloroplasts by epifluorescence and electron microscopy, and consequently revealed a novel role for *EGY1* function in the control of epidermal chloroplast development. Chloroplast distribution in the leaf epidermis has been documented in the previous literature, and this fact has been described and argued by several landmark studies [2,4,5,26,34,77]. To date, however, systematic analyses of the phenotypes of mesophyll, pavement, and guard cell chloroplasts in the mesophyll chloroplast biogenesis mutants, have not been performed, with a few exceptions. Here, we report that *EGY1* encodes a critical factor involved in the biogenesis of epidermal chloroplasts—in particular the differentiation of guard cell chloroplasts—although mutations in *EGY1* permitted the development of mesophyll chloroplasts in mutant leaves [49]. The previous finding that *egy1* mutants possess chlorophyll-less amyloplasts in the hypocotyl endodermis [78], together with our current comparative analysis of epidermal chloroplast formation in *egy1* and *sig2-2* mutants, suggests that *EGY1* has a greater impact on the formation of non-mesophyll chloroplasts than mesophyll chloroplasts in green tissues of *Arabidopsis* plants. While a vast number of studies have identified many organ- or tissue-specific chloroplast biogenesis mutants or cultivars based on leaf coloration phenotypes [55,79,80,81], these leaf phenotypes were visually dissected on a two-dimensional scale in most cases. Nevertheless, tissue-specific defects in chloroplast formation due to a particular gene mutation can occur along the z-axis of leaf blades involving the leaf epidermis.

The results of the current study raise many questions: (i) How is the development of epidermal chloroplasts in guard cells and pavement cells spatially controlled during leaf formation? (ii) How and why do *EGY1* mutations affect the differentiation of leaf epidermal chloroplasts, especially guard cell chloroplasts, more severely than that of mesophyll chloroplasts? (iii) What is the most crucial factor controlling chloroplast development in non-mesophyll tissues? (iv) What is the physiological significance of epidermal chloroplasts, besides their involvement in leaf coloration (e.g., [77]), cuticle development (e.g., [82]), stomatal opening and closing (e.g., [23,39,83,84]), abiotic stress signaling (e.g., [85]), and plant defense (e.g., [86,87,88])? (v) To what extent are mechanisms underlying the development of mesophyll and non-mesophyll chloroplasts conserved or divergent among plant species? These questions should be addressed in future studies using various experimental approaches.

The current knowledge of leaf development may provide clues for tackling these questions. To answer question (i), approximately 50% of the pavement cells in mature leaves are predicted to be derived from the stomatal lineage [72]. For instance, during the proliferation of meristemoids, asymmetric division supplies both meristemoids and pavement cells as daughter cells, and such stomatal lineage-derived pavement cells might differ from the other pavement cells in terms of the control of epidermal chloroplast development. Various phenotypic characteristics of *egy1* pavement cell chloroplasts could thus be attributed to the developmental heterogeneity of pavement cells. We are currently investigating this possibility using the leaves of *egy1* mutants. Additionally, regarding question (ii), data publicly available in the eFP browser [89] show that *EGY1* mRNAs accumulate to higher levels in leaf guard cells than in mesophyll cells, indicating that the higher concentration of the EGY1 protein in guard cell chloroplasts might reflect its greater physiological significance compared with that of mesophyll cell chloroplasts. Nevertheless, the substrate of the EGY1 protease remains unknown, and substantial experimental evidence would be required to support the above notion.

From a morphological point of view, unusual thylakoid structures found in Ar50-33-pg1 and *egy1-4* mutants deserve extensive arguments based on comparisons with previously reported mutant or wild-type chloroplast research. For example, the structural features of thylakoids in *egy1* mutant epidermal chloroplasts, i.e., single-layered, spiral, and circular, were similar to those of cotyledon mesophyll chloroplasts, which differentiated from etioplasts immediately after light irradiation [90]. Furthermore, thylakoids in the leaf mesophyll chloroplasts of the *constitutively photomorphogenic 1-6* (*cop1-6*) *Arabidopsis* mutant bear a significant resemblance to those in the mesophyll chloroplasts of *egy1* mutants [91]. These data suggest that thylakoids in *cop1-6*, Ar50-33-pg1, and *egy1-4* mutants exhibit a common growth arrest phenotype during chloroplast differentiation. According to this assumption, *EGY1* might be critical for the biogenesis of thylakoid membranes in the early to middle stages of chloroplast differentiation, although the mechanisms underlying the formation of spiral, circular, and interconnected thylakoids remain unknown. Additionally, it is possible that grana degeneration, as found in the mesophyll chloroplasts of *egy1* mutants [49], contributes to the thylakoid architecture of guard cell and pavement cell chloroplasts in the leaf epidermis. Given the increased accumulation of plastoglobules in both epidermal cell types in the leaves of 10-day- and 3-week-old plants (Figure 7B,D, Figure 8B and Figure 9), it is possible that the balance between thylakoid biogenesis and degeneration processes determines the terminal phenotype of leaf epidermal chloroplasts in *egy1* mutants.

## 4. Materials and Methods

### 4.1. Plant Materials and Growth Condition

*Arabidopsis thaliana* accession Columbia (Col) was used as the wild type in this study. All mutants used in this study were also generated in the Col genetic background. Two ^40^Ar^17+^-irradiated mutants, Ar50-33-pg1 [49] and *egy1-4* (originally Ar-28-pg1) [50], were generated previously. Two chloroplast biogenesis mutants, *sig2-2* [53,54] and *im-1* [37], were provided by RIKEN BRC (originally SALK_006646C [92]; psy01036; Tsukuba, Japan) and Nottingham Arabidopsis Stock Centre (CS3639; Leicestershire, UK), respectively. A fluorescent transgenic line, FL6-5, expressing a plastid stroma-targeted YFP and nucleus-targeted CFP under the control of the CaMV*35S* promoter, was previously described [32]. Seeds of all genotypes were surface-sterilized and sown on Jiffy-7 (AS Jiffy Products, Stange, Norway) or on MS agar media [93] (Wako, Osaka, Japan) supplemented with Gamborg’s B5 vitamins and 3% (*w*/*v*) sucrose. All plants were grown under a long-day photoperiod, as described previously [32].

### 4.2. Microscopy

To observe the live tissues of *Arabidopsis* plants, epifluorescence microscopy and fluorescence stereomicroscopy were performed as described previously [31,33]. To perform epifluorescence microscopy, either excised organs or epidermal peels obtained from the abaxial surface of leaves or cotyledons were mounted on glass slides and observed under an Olympus IX71 microscope (Tokyo, Japan). Optical filters and objective lenses used for the detection of chlorophyll autofluorescence, and GFP and YFP signals, have been described previously [33]. To perform fluorescence stereomicroscopy, whole seedlings or excised organs were observed under a Leica MZ10 F microscope (Heidelberg, Germany). Standard band-path and long-path filters and objective lenses (0.63× and 1.60×; Leica Microsystems, Wetzlar, Germany) were used to detect chlorophyll autofluorescence and GFP and YFP signals.

The ultrastructure of leaf epidermal plastids was analyzed by TEM. Briefly, leaf samples were collected and subjected to primary chemical fixation, as described previously [49]. Subsequent processes of secondary fixation, dehydration, and preparation of ultrathin sections were performed by Tokai Electron Microscopy Inc. (Nagoya, Japan), as described previously [27].

The obtained digital gray-scale images were processed using Photoshop (Adobe Systems, San Jose, CA, USA) and ImageJ (v1.48, National Institute of Health, Bethesda, MD, USA) to obtain pseudo-colored and merged images. In each panel of Figure 1, Figure 2, Figure 3, Figure 4, Figure 5 and Figure 6 and Supplementary Figures S1–S5, GFP and chlorophyll fluorescence images were taken under the same excitation and detection conditions, respectively.

### 4.3. Generation of Transgenic Arabidopsis Lines with GFP-Labeled Plastids in the Leaf Epidermis

To specifically label epidermal plastids in the shoot tissues of *A. thaliana*, the stroma-targeted synthetic (S65T) GFP [59] was used as a reporter. The N-terminal fusion of GFP with the SIG6 (formerly known as SigF [60]) TP was expressed in *Arabidopsis* plants under the control of the *PDF1* promoter [61,62]. To construct the *PDF1*p::*TP_SIG6_-GFP* plasmid, a 1.5 kb sequence upstream of the *PDF1* gene was amplified from the genomic DNA of Col plants by PCR using two gene-specific primers, PDF1-4 (TCCTG*AAGCTT*TGAATTTAAAACATTTTTTTTTATATATATAG) and PDF1-5 (ATACC*CTCGAG*TTTATGAGAATTCACTGAGATTCAGAGAG) (italicized nucleotides represent restriction sites), and PrimeStar DNA polymerase (Takara, Osaka, Japan). PCR was conducted on a thermal cycler (model T100; Bio-Rad, Hercules, CA, USA) under the following conditions: 45 cycles of denaturation at 95 °C for 25 s, annealing at 60 °C for 30 s, and extension at 72 °C for 1 min 50 s. The PCR product was digested with *Hin*dIII and *Xho*I restriction endonucleases and then ligated into the pF-TP-GFP vector (provided by Drs. K. Tanaka and Y. Niwa) [60], which were predigested with *Hin*dIII and *Sal*I to delete the CaMV*35S* promoter from the CaMV*35S*p::*TP_SIG6_-GFP*::*NOS*t cassette. A 2.8 kb *Hin*dIII-*Pst*I fragment of the resulting plasmid, corresponding to the *PDF1*p::*TP_SIG6_-GFP* cassette, was introduced into the pSMAB-Z1TP-sC vector [17] (the original binary vector was provided by Dr. H. Ichikawa) by simultaneously deleting the *35S*p::*TP_FtsZ1_-CFP* cassette. The resulting binary vector, pSMAB-PDF1p2-FTP-GFP (13.0 kb), was transformed into *Agrobacterium tumefaciens* strain C58 by the freeze–thaw method. *Agrobacterium*-mediated transformation of wild-type (Col) plants was performed using the floral dip method [63]. Over 800 transformed (T_1_) seedlings were selected on MS media containing 10 µg/mL bialaphos, and 15 T_1_ seedlings were selected to examine the strength, localization, and stability of the GFP signal by fluorescence stereomicroscopy. Finally, one stable transgenic line, designated as FG13-16, was chosen for the microscopic analysis of epidermal chloroplasts.

## Figures and Tables

**Table 1:** Plastid number in leaf stomatal guard cells of wild-type plants and mutant lines expressing *PDF1*p::*TP_SIG6_-GFP*.

Plant		Wild Type (FG13-16)	Ar50-33-pg1	*egy1-4*
Chloroplast	Mean ± SD	7.3 ± 1.1	7.6 ± 1.1	7.8 ± 1.2
number	Max	10	10	11
per stoma	Min	5	6	5
(GC pair)	n	50	50	50
per GC	Mean ± SD	3.6 ± 0.8	3.8 ± 0.8	3.9 ± 0.8
	Max	6	6	6
	Min	2	2	2
	n	100	100	100

## Data Availability

Data is contained within the article or supplementary material.

## Supplementary Materials

The following are available online at https://www.mdpi.com/article/10.3390/plants10061254/s1. Figure S1: Fluorescence microscopy analysis of the epidermis of fully expanded leaves of wild-type and mutant plants; Figure S2: Phenotypic characterization of the F_1_ progeny of Ar50-33-pg1 and *egy1-4* by fluorescence microscopy; Figure S3: Phenotypic characterization of the T-DNA insertion mutant *egy1-2* and the F_1_ progeny of *egy1-2* and *egy1-4*. Figure S4: Generation of transgenic *Arabidopsis* lines expressing *PDF1*p::*TP_SIG6_-GFP*; Figure S5: Characterization of the *PDF1*p::*TP_SIG6_-GFP* transgenic line FG13-16; Figure S6: Fluorescence microscopy of leaf guard mother cells in mutant plants expressing *PDF1*p::*TP_SIG6_-GFP*; Figure S7: TEM analysis of mesophyll and pavement cells in an expanding leaf of Ar50-33-pg1 mutant plants; Figure S8: TEM analysis of guard cells in fully expanded leaves of wild-type (Col) and Ar50-33-pg1 mutant plants. Table S1: Segregation analysis and allelism test of *egy1-4*.
