# Supplementary material for: Arabidopsis EGY1 Is Critical for Chloroplast Development in Leaf Epidermal Guard Cells
Source: Plants (Basel). 2021 Jun 21;10(6):1254. doi: 10.3390/plants10061254 (PMC8235790; doi:10.3390/plants10061254)
Supplement: Supplementary file 1 [file plants-10-01254-s001.zip › plants-1230604-SI.pdf]

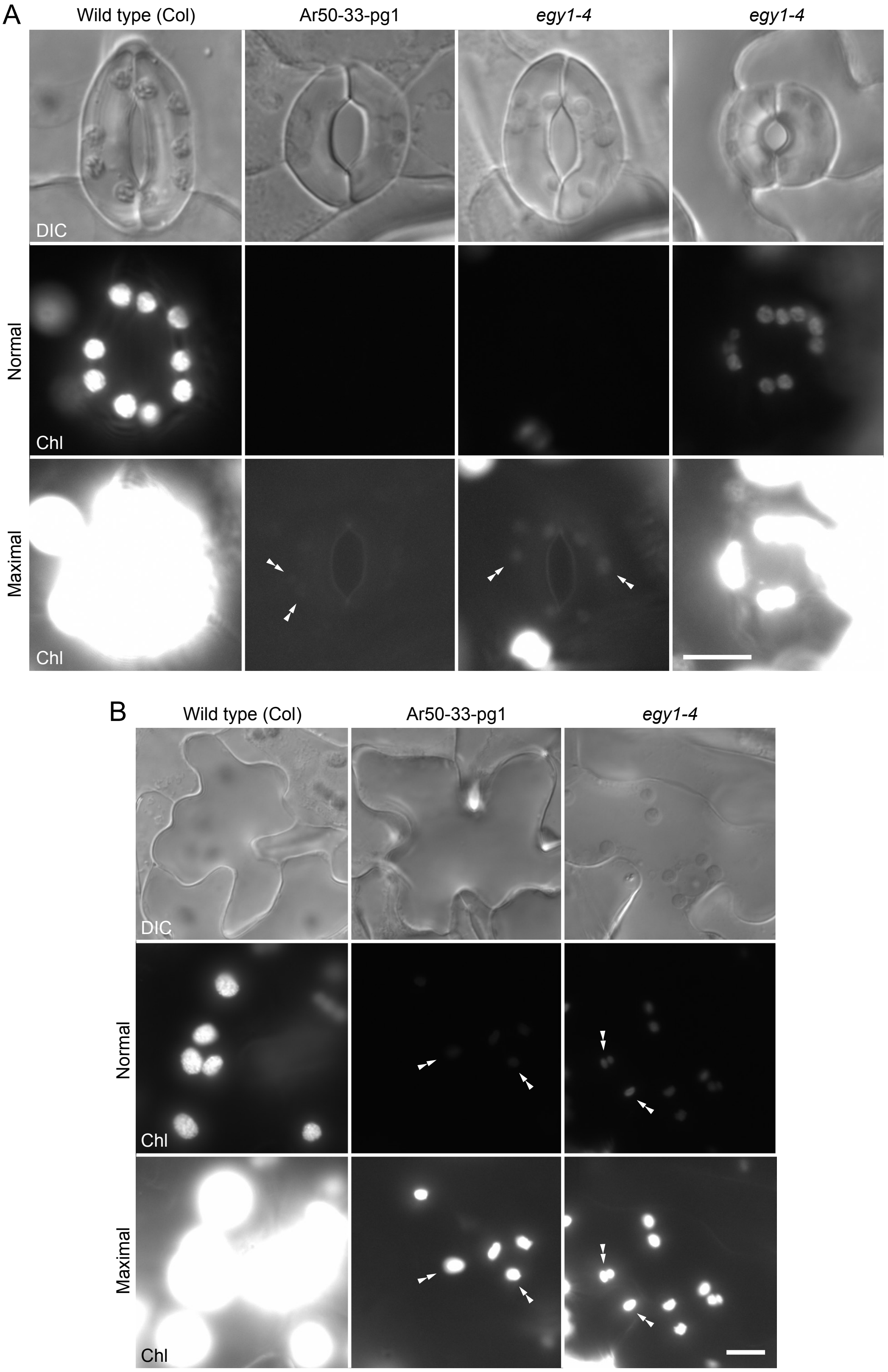

Figure S1. Fluorescence microscopy analysis of the epidermis of fully expanded leaves of wild type and mutant plants. (**A**,**B**) Differential interference contrast (DIC) and chlorophyll autofluorescence (Chl) images of guard cells (**A**) and pavement cells (**B**) in gray scale. Chl images were taken at the normal settings (camera gain: 50; exposure time: 1.0 s; excitation strength: 12%) and maximal settings (camera gain: 250; exposure time: 2.0 s; excitation strength: 100%). Double arrowheads indicate chloroplasts. Scale bar = 10 μm (**A**,**B**). This figure is supplemental to Figure 1.

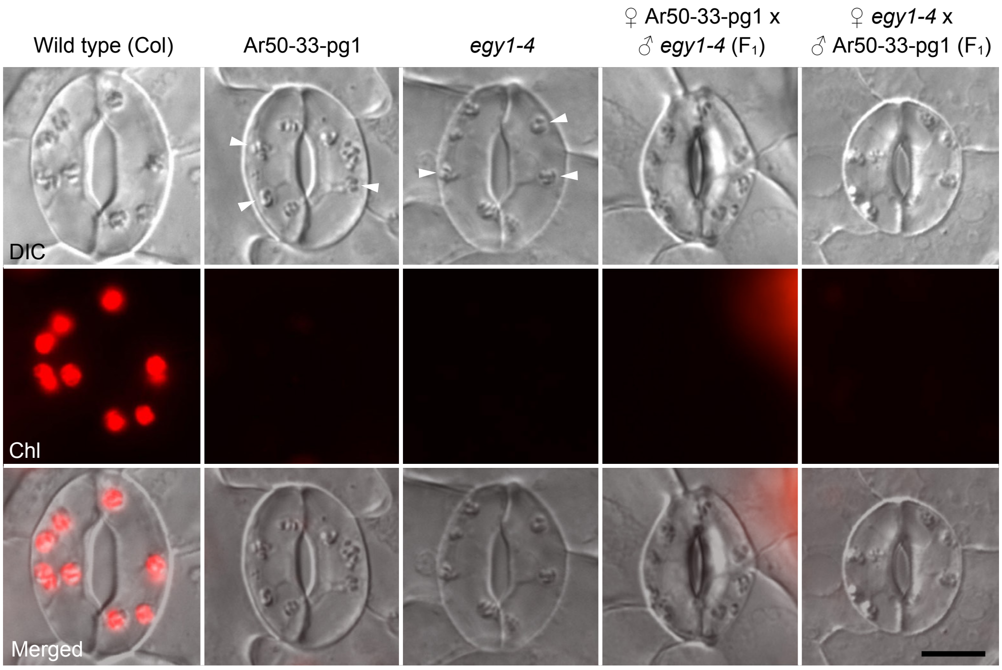

Figure S2. Phenotypic characterization of the F_1 progeny of Ar50-33-pg1 and *egy1-4* by fluorescence microscopy. DIC, chlorophyll autofluorescence (Chl), and merged images of the abaxial leaf epidermal peels obtained from the third and fourth expanding leaves of 2-week-old seedlings. Arrowheads indicate putative chloroplasts or plastids. Scale bar = 10 μm.

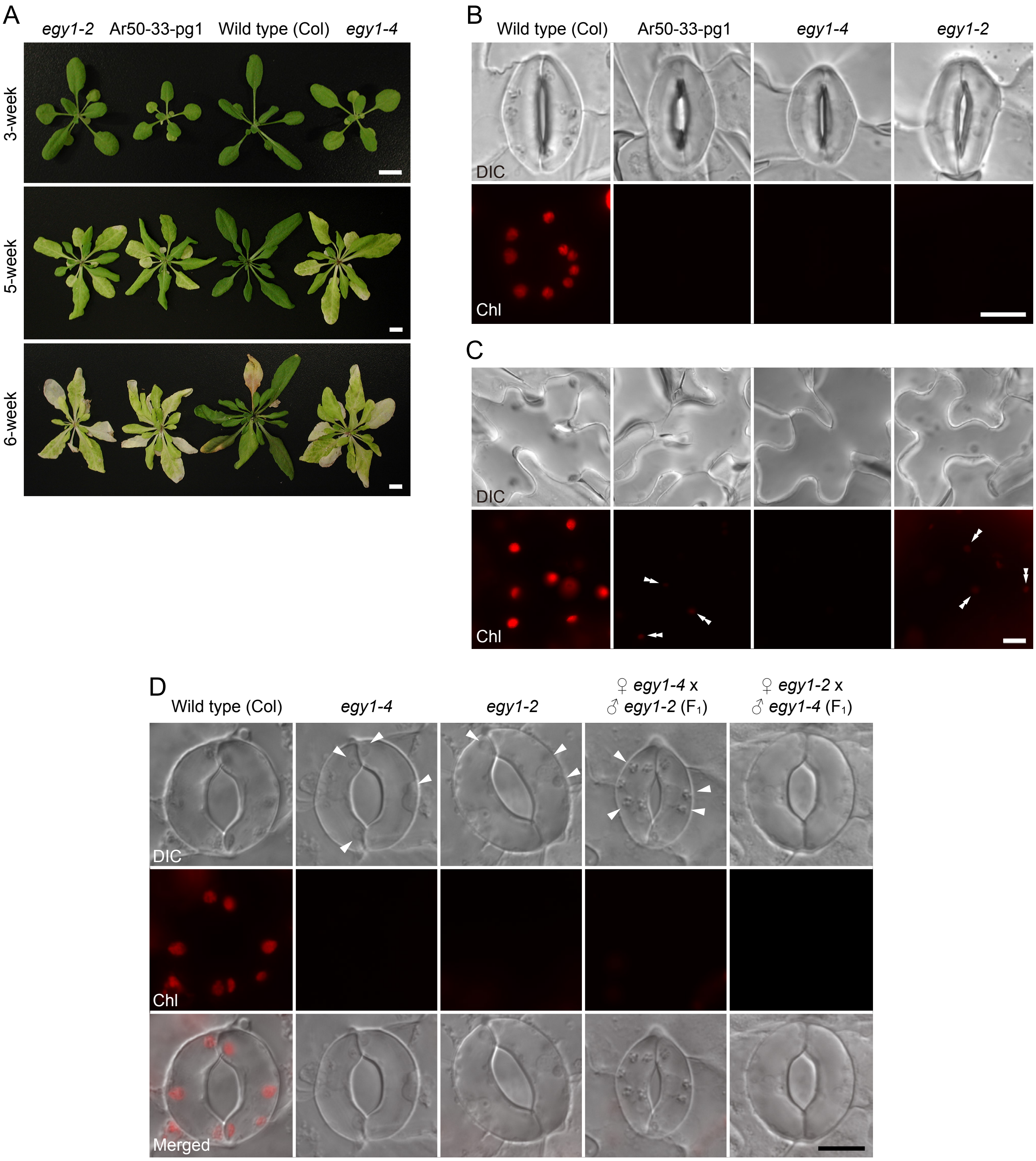

Figure S3. Phenotypic characterization of the T-DNA insertion mutant *egy1-2* and the F_1 progeny of *egy1-2* and *egy1-4*. (**A**) Rosette morphology of 3-week-, 5-week-, and 6-week-old wild type and mutant plants. Stems were removed just prior to image capture. The image of 6-week-old plants was originally published by Sanjaya et al. (2021) and reproduced. (**B–D**) Fluorescence microscopy of leaf epidermis of the wild type and mutant leaves. DIC, chlorophyll autofluorescence (Chl), and merged images of guard cells (**B**,**D**) and pavement cells (**C**) of the abaxial leaf epidermal peels obtained from the primary leaves of 3-week- (**B**,**C**) and 2-week-old (**D**) seedlings. Arrowheads indicate putative chloroplasts or plastids. Double arrowheads indicate chloroplasts. Scale bars = 1 cm (**A**) and 10 μm (**B–D**).

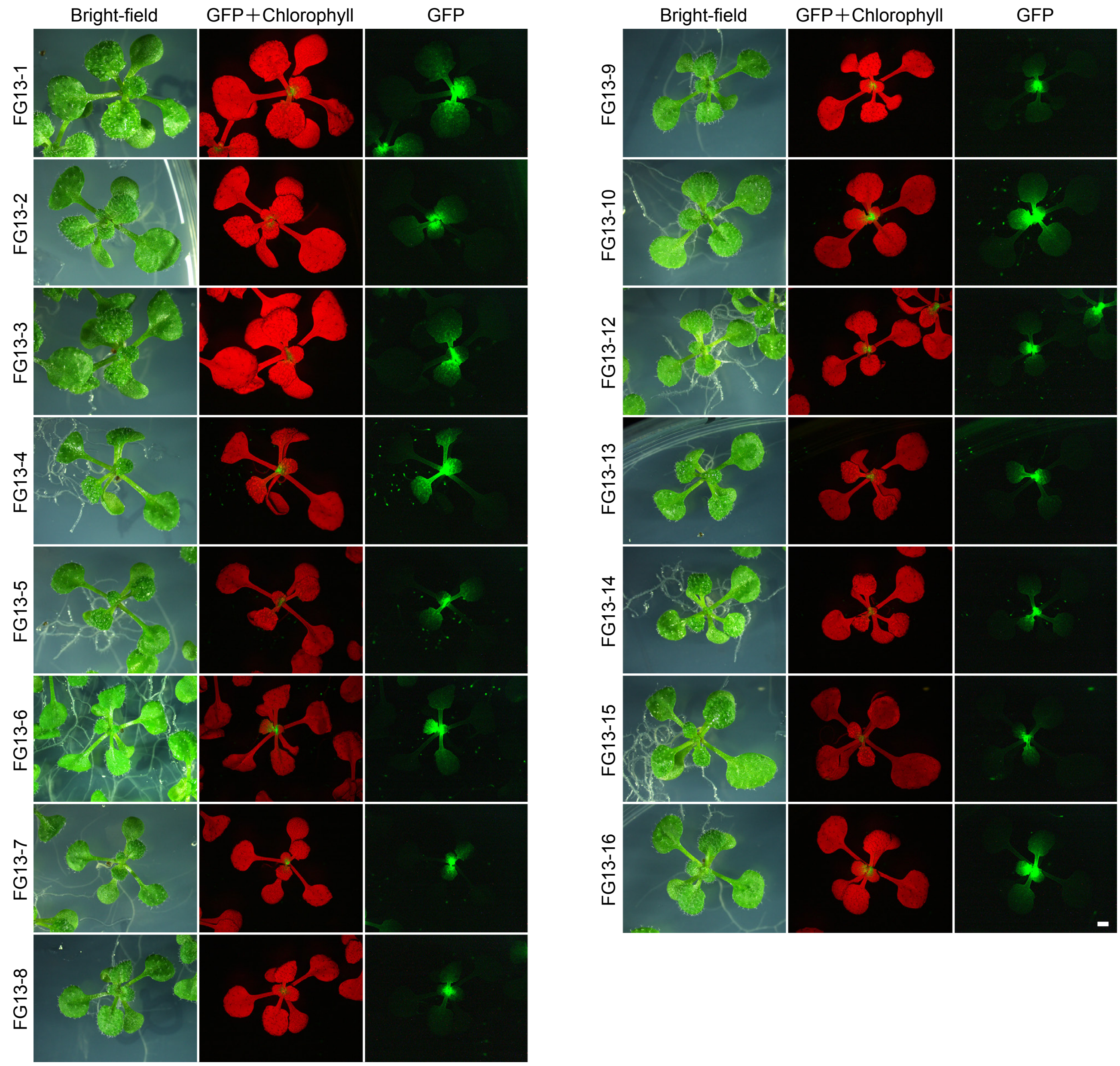

Figure S4. Generation of transgenic *Arabidopsis* lines expressing *PDF1*p::*TP*$_{SIG6}$-*GFP*. Images showing the bright-field view and GFP signal, which was detected using a long-path filter (GFP+Chl) or a band-path filter, in 2-week-old transgenic seedlings in the T_2 generation are shown. Scale bar = 1 mm. This figure is supplemental to Figure 3.

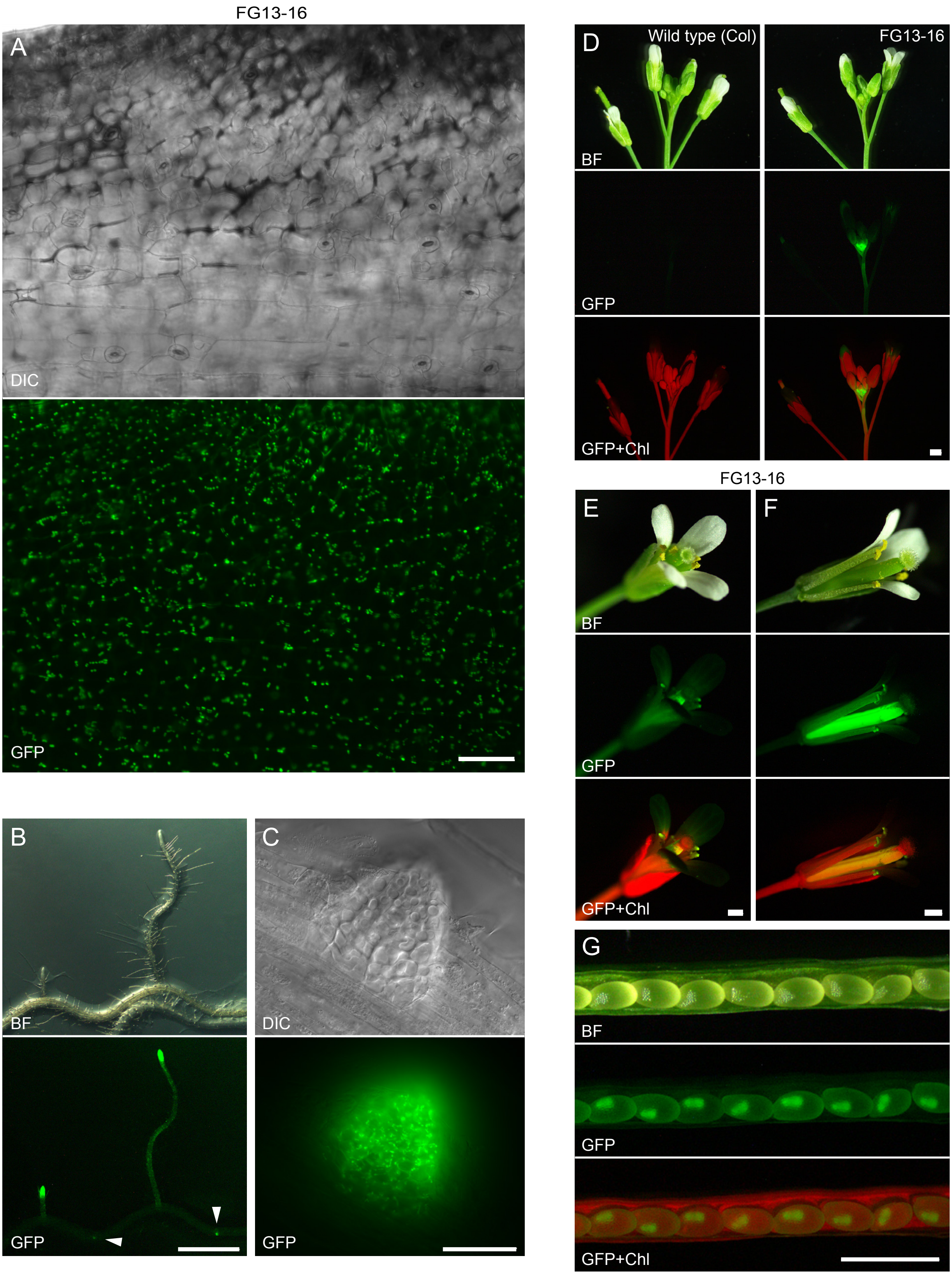

Figure S5. Characterization of the *PDF1*p::TP_{SIG6}-*GFP* transgenic line FG13-16. (**A–G**) Bright-field (BF), DIC, and GFP images of intact developing leaf (**A**), lateral root (**B**), lateral root primordium (**C**), inflorescence (**D**), intact flower (**E**), dissected flower (**F**), and silique and ovules (**G**). Arrowheads in (**B**) indicate primordia emerging from the main root. GFP images were captured using a band-path filter (GFP) or a long-path filter (GFP+Chl). Scale bars = 50 μm (**A**), 0.5 mm (**B**, **E–G**), 20 μm (**C**), and 1 mm (**D**). This figure is supplemental to Figure 3.

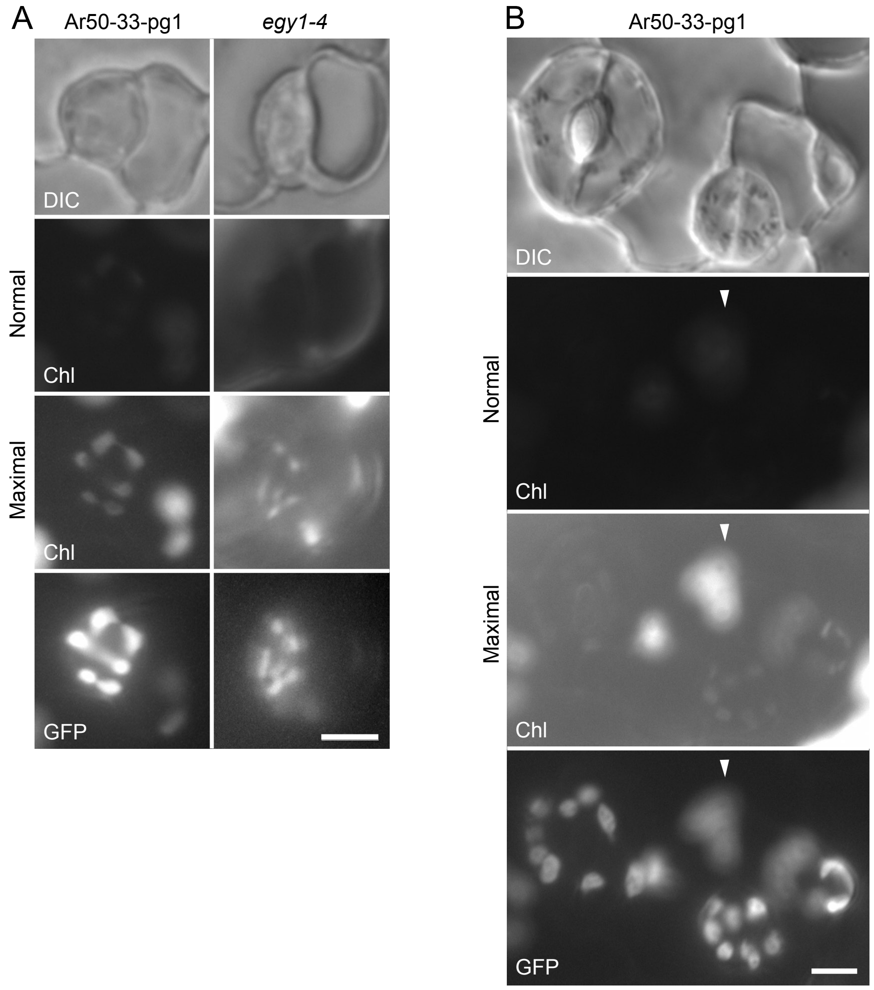

Figure S6. Fluorescence microscopy of leaf guard mother cells in mutant plants expressing *PDF1*p::*TP*$_{SIG6}$-*GFP*. DIC, chlorophyll autofluorescence (Chl), and GFP images are shown in gray scale. Chl images were taken at the normal settings (camera gain: 50; exposure time: 1.0 s; excitation strength: 12%) and maximal settings (camera gain: 250; exposure time: 2.0 s; excitation strength: 100%). Arrowheads in (**B**) correspond to those in Figures 4 and 6, and indicate pavement chloroplasts. Scale bar = 0.5 μm (**A**) and 5 μm (**B**). This figure is supplemental to Figure 6.

Ar50-33-pg1

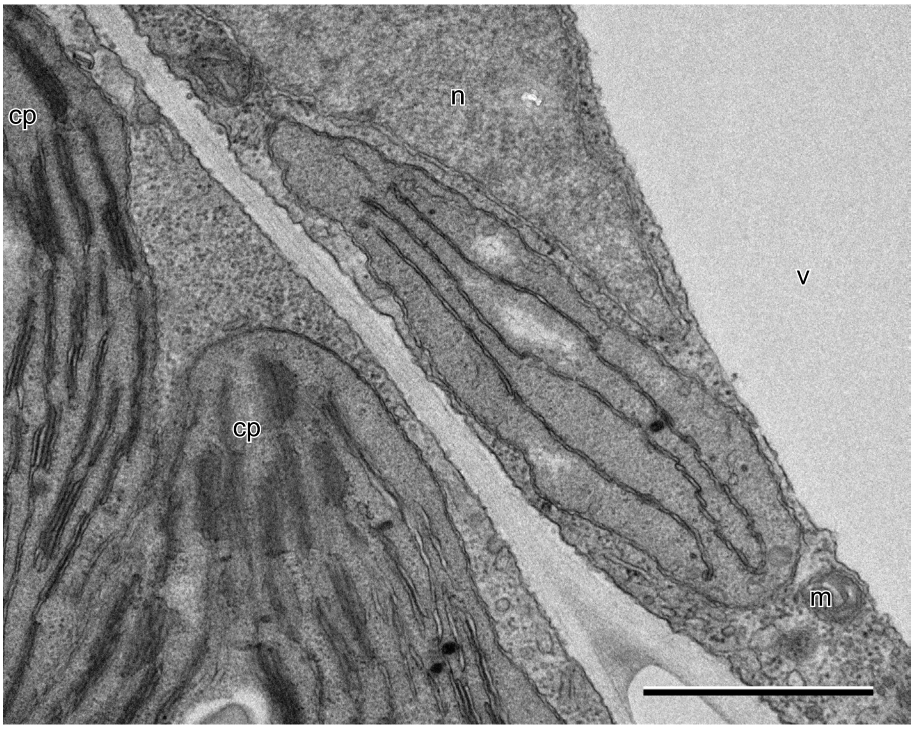

Figure S7. TEM analysis of mesophyll and pavement cells in an expanding leaf of Ar50-33-pg1 mutant plants. Abbreviations: cp, mesophyll cell chloroplast; m, mitochondrion; n, pavement cell nucleus; v, vacuole. Scale bar = 1 μm. This figure is supplemental to Figure 8.

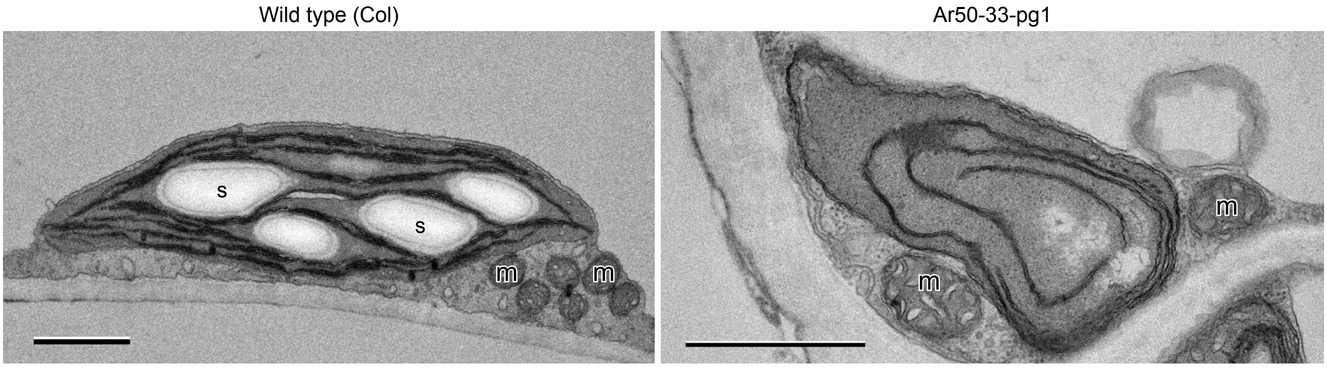

Figure S8. TEM analysis of guard cells in fully expanded leaves of wild-type (Col) and Ar50-33-pg1 mutant plants. Abbreviations: m, mitochondrion; s, starch grain. Scale bar = 1 μm. This figure is supplemental to Figure 9.

plants

MDPI

Table S1. Segregation analysis and allelism test of *egy1-4*. Phenotypes of F_1 and F_2 seedlings after growth on MS media for 14 days.

Cross	Generation	Number of Plants			Expected ratio (Normal : Pale green)	χ^2 value
		Total	Normal	Pale green		
♂*egy1-4** × ♀Col	F_1	15	15	0	1 : 0	0.00
♂*egy1-4** × ♀Col	F_2	390	294	96	3 : 1	0.03
♂*egy1-4** × ♀*egy1-2**	F_1	>20	0	>20	0 : 1	0.00
♂*egy1-2** × ♀*egy1-4**	F_1	>20	0	>20	0 : 1	0.00
♂FG13-16 × ♀*egy1-4**	F_1	50	50	0	1 : 0	0.00

*Plants with homozygous *egy1* mutations were used for crossing.
